# Cysts of the Snow Alga *Chloromonas krienitzii* (Chlorophyceae) Show Increased Tolerance to Ultraviolet Radiation and Elevated Visible Light

**DOI:** 10.3389/fpls.2020.617250

**Published:** 2020-12-17

**Authors:** Lenka Procházková, Daniel Remias, Wolfgang Bilger, Heda Křížková, Tomáš Řezanka, Linda Nedbalová

**Affiliations:** ^1^Faculty of Science, Charles University, Prague, Czechia; ^2^School of Engineering, University of Applied Sciences Upper Austria, Wels, Austria; ^3^Botanical Institute, Christian-Albrechts-University Kiel, Kiel, Germany; ^4^Institute of Microbiology, The Czech Academy of Sciences, Prague, Czechia

**Keywords:** snow algae, cysts, polyunsaturated fatty acids, photosynthesis, astaxanthin, chlorophyll fluorescence, UV-B radiation, UV-A radiation

## Abstract

Melting mountainous snowfields are populated by extremophilic microorganisms. An alga causing orange snow above timberline in the High Tatra Mountains (Poland) was characterised using multiple methods examining its ultrastructure, genetics, life cycle, photosynthesis and ecophysiology. Based on light and electron microscopy and ITS2 rDNA, the species was identified as *Chloromonas krienitzii* (Chlorophyceae). Recently, the taxon was described from Japan. However, cellular adaptations to its harsh environment and details about the life cycle were so far unknown. In this study, the snow surface population consisted of egg-shaped cysts containing large numbers of lipid bodies filled presumably with the secondary carotenoid astaxanthin. The outer, spiked cell wall was shed during cell maturation. Before this developmental step, the cysts resembled a different snow alga, *Chloromonas brevispina*. The remaining, long-lasting smooth cell wall showed a striking UV-induced blue autofluorescence, indicating the presence of short wavelengths absorbing, protective compounds, potentially sporopollenin containing polyphenolic components. Applying a chlorophyll fluorescence assay on intact cells, a significant UV-A and UV-B screening capability of about 30 and 50%, respectively, was measured. Moreover, intracellular secondary carotenoids were responsible for a reduction of blue-green light absorbed by chloroplasts by about 50%. These results revealed the high capacity of cysts to reduce the impact of harmful UV and high visible irradiation to the chloroplast and nucleus when exposed at alpine snow surfaces during melting. Consistently, the observed photosynthetic performance of photosystem II (evaluated by fluorometry) showed no decline up to 2100 μmol photons m^–2^ s^–1^. Cysts accumulated high contents of polyunsaturated fatty acids (about 60% of fatty acids), which are advantageous at low temperatures. In the course of this study, *C. krienitzii* was found also in Slovakia, Italy, Greece and the United States, indicating a widespread distribution in the Northern Hemisphere.

## Introduction

Long-lasting, melting snow is an extremophilic habitat for specialised phototrophic eukaryotes (Hoham and Remias, 2020). Cryoflora represents the primary producers in an ecosystem containing heterotrophic members like bacteria, ciliates, mites, rotifers, tardigrades, springtails in combination with fungi like chytrids (Yakimovich et al., 2020). During the melting period, snow algae cause the macroscopic phenomenon of coloured snow. Such blooms frequently occur in combination with abundant intracellular secondary carotenoids, altering the snow colour from green to red. This has an impact on snowmelt due to a significant albedo reduction, as shown in Polar (Onuma et al., 2016; Gray et al., 2020; Khan et al., 2020) and Alpine regions (Di Mauro et al., 2020).

Using state of the art morphological and molecular protocols, the number of recognised species causing snow blooms has increased during the last decade (e.g., Matsuzaki et al., 2019). The majority belongs to the green algal genus *Chloromonas*, and its ability of snow colonisation is based on a life cycle including sensitive, migrating flagellates deeper in the snow and a transformation into robust, immotile cyst stages that prevail at the surface (Hoham and Remias, 2020). Generally, knowledge about the geographic distribution or the ecophysiological preferences of these cryoflora microbes is limited. Some have been studied more extensively, such as the red snow causing *Chlainomonas* sp. (Remias et al., 2016; Procházková et al., 2018a; the first record from Antarctica in Luo et al., 2020) or *Sanguina nivaloides* (formerly assigned to *Chlamydomonas nivalis*) (Procházková et al., 2019a). There are not many works about snow dwelling *Chloromonas* that cause colours other than red (Hoham et al., 2006; Remias et al., 2010; Procházková et al., 2019b).

In this study, the ecophysiology of *Chloromonas krienitzii* (Chlorophyceae) was investigated. Specimens were collected in several alpine sites of Europe and North America, where cyst stages were responsible for orange blooms during summer. Historically, this species was one of several lineages formerly assigned to *Chloromonas brevispina*, which comprised characteristic cysts with cells possessing uniformly distributed spiky surface structures (Hoham et al., 1979). With samples from Japan, Matsuzaki et al. (2015) described an independent taxon out of this group based on morphological data and the multigene phylogeny of the vegetative strain. Although this species was recently detected by environmental sequencing in British Columbia (Canada) (Engstrom et al., 2020), its overall geographic distribution is still unexplored.

Cells of *C. krienitzii* were characterised using multiple approaches to understand strategies of adaptation to the harsh habitat. In a first step, the field collected cells were determined to the species level using the hypervariable ITS2 rDNA marker (barcoding). Second, since several details of the life cycle including cleavage of cysts into flagellates for this alga were unknown, thus hampering determination efforts by light microscopy of environmental samples, the putative life cycle was reconstructed. Third, since cysts are subject to high irradiation at the snow surface, the light-dependent photobiology was evaluated. Fatty acid and pigment profiles of the cysts complement the dataset, as these compounds are regarded as important players for low temperature and high irradiation adaptation (Leya, 2013, 2020). Finally, by applying a chlorophyll fluorescence assay to intact cells, the capacity for screening the chloroplast in the cysts against harmful radiation by UV absorbing carotenoids was measured to reveal the capability to reduce the impact of this irradiation.

## Materials and Methods

### Field Sampling

Table 1 shows the snow sampling sites in the Rocky Mts. (Colorado, United States), the Sarntal Alps (South Tyrol, Italy), High Tatra Mts. (Slovakia, Poland) and North Pindus (Greece) (Supplementary Figure 1). With a field microscope, virtually monospecific blooms (LP05, WP191, Pind19) were harvested according to Procházková et al. (2019a). The unialgal spot of 1SAR was collected without previous light microscopy in the field. Additionally, three mixed algal snow communities (Saddle2, WP189, WP190) were included in this study to track the geographical distribution of the target species. Harvested snow was placed in 5 L buckets (LP05) or in 50 mL centrifugation tubes (all the other samples). Supplementary, a 10 mL subsample of LP05 was fixed immediately after harvest by a drop of acidic lugol solution (acetic acid). Samples LP05 and WP191 were used for cultivation assay. Samples LP05, WP191 and 1SAR were a subject of Sanger sequencing. Furthermore, sample LP05 was also used in other subsequent analysis (electron microscopy, photosynthesis measurements, UV and blue light transmittance protocol, analyses of pigment and fatty acid profiles). Prior to photosynthesis measurements, snow with live cells was melted gently in darkness overnight at 4–5°C. Electrical conductivity (EC) and pH of the meltwater were obtained with WTW Instruments (Cond 340i and Inolab, Germany) or with HANNA (Combo EC, ftb Romania). The snow water equivalent at the sampling site (SWE; referred to as “snow water content” in the following reference) was carried out as described previously (Procházková et al., 2018b).

**TABLE 1:** *Chloromonas krienitzii* sample codes, collection date, sampling site, elevation (metres a.s.l.) and geographic position (GPS).

**Sample**	**Date**	**Location**	**Altitude**	**GPS**	**#**
Saddle2	July 8, 2017	Niwot Ridge, Front Range, Rocky Mts, Colorado, United States	3540	N40° 3.326′ W105° 35.265′	1
1SAR	July 15, 2013	Wannser Joch, Sarntal Alps, South Tyrol (BZ), Italy	2244	N46° 47.867′ E11° 21.697′	2
LP05	June 14, 2017	Valley Za Mnichem, High Tatras, Poland	1862	N49° 11.654′ E20° 3.168′	3
WP189	June 17, 2018	Next to Modré Lake shore, High Tatras, Slovakia	2177	N49° 11.559′ E20° 11.165′	4
WP190	June 17, 2018	At the bottom of Dolinka pod Sedielkom, High Tatras, Slovakia	2114	N49° 11.41′ E20° 11.27′	5
WP191	June 17, 2018	On a shore of Prostredné Sivé Lake, High Tatras, Slovakia	2017	N49° 11.016′ E20° 10.483′	6
Pind19	July 1, 2019	Next to a hiking trail, North Pindus, Greece	2376	N40° 5.153′ E20° 55.302′	7

*The numbers indicate sampling sites at the map of Supplementary Figure 1.*

### Cultivation Assay

A feature of very low electrical conductivities recorded in the field was reproduced at laboratory conditions for cultivation. As long as the field cysts did not start to cleave, they were incubated in deionised water. In detail, for gaining unialgal strains out of field blooms to study the life cycle, subsamples of LP05 and WP191 containing sedimented cysts were put into sterile 2 mL cryotubes and the meltwater replaced with deionised water. For induction of germination of the cysts, the cells were kept at 1°C during the day (14 h) resp. −1°C during the night (10 h) in a Percival LT-36VL (CLF Plant Climatics, Wertingen, Germany). The light intensity generated by fluorescent tubes was approximately 40–70 μmol PAR m^–2^ s^–1^. Subsequently, flagellates were transferred from the supernatant into liquid 0.6 N Bold’s Basal Medium (BBM) (Bischoff and Bold, 1963) and irradiance was kept at 20–30 μmol PAR m^–2^ s^–1^.

### Light and Electron Microscopy

Light microscopy was performed with an Olympus BX43 at 1000× magnification using oil immersion, equipped with an Olympus DP27 camera (Olympus Europe SE, Hamburg, Germany), or with a Nikon Eclipse 80i with Nikon DS-5M camera (Nikon Instruments Europe, BV). Scanning and transmission electron microscopy (SEM and TEM) were carried out with sample LP05 as described previously (Procházková et al., 2018b), with the minor exception that for TEM only cells previously fixed by acidic lugol solution (acetic acid) were used. The autofluorescence of cell walls was studied using Nikon Eclipse 80i (objective Plan Apo VC 100 × 1.40, camera DS 5 M; Nikon Instruments, Amsterdam, Netherlands) equipped with a DAPI-filter (excitation = 340–380 nm, emission = 435–485 nm). Chloroplast shapes were visualised, by exposing them to excitation = 460–500 nm and emission 600 nm.

### Isolation of DNA, PCR, Sequencing, ITS2 rRNA Secondary Structure Prediction

DNA isolation (sample LP05) was carried out with a DNeasy Plant Mini Kit (Qiagen, Germany), as in Procházková et al. (2018b). If less than 20 mg wet biomass was available (samples WP191 and 1SAR), DNA was extracted using the Instagene Matrix Kit (Bio-Rad Laboratories, Hercules, CA, United States) according to Remias et al. (2016). The internal transcribed spacer region 2 (ITS2 rDNA) was amplified from DNA isolates by polymerase chain reaction (PCR) using existing primers of SSU (CTGCGGAAGGATCATTGATTC) and LSU (AGTTCAGCGGGTGGTCTTG) (Piercey-Normore and DePriest, 2001). Amplification reactions were described in Procházková et al. (2018b). PCR products were purified and sequenced using an Applied Biosystems automated sequencer (ABI 3730xl) at Macrogen Europe (Amsterdam, Netherlands). The obtained sequences of *C. krienitzii* were submitted to NCBI Nucleotide sequence database (accession numbers: MW136658 for LP05–partial 18S rDNA + ITS1 rDNA + ITS2 rDNA + partial 28S rDNA; MW139361 for WP191–ITS2 rDNA; MW139362 for 1SAR–ITS2 rDNA). Methods for annotation and prediction of the secondary structure of the nuclear rDNA ITS2 region follow those described in a previous study (Remias et al., 2020).

### Photosynthesis

*In vivo* chlorophyll fluorescence parameters were measured with a pulse–amplitude modulated fluorometer (PAM 2500, Heinz Walz GmbH, Germany) in a 0.6 mL chamber and cooled in an ice bath to approximately 2°C. To obtain the relative electron transport rates (rETRs), the apparent quantum yield for electron transport (alpha) and the light saturation point I_k_, cells were exposed to photon flux densities (PFD) of 5, 9, 34, 67, 104, 201, 366, 622, 984, 1389, 1666, and 2018 μmol photons m^–2^ s^–1^ for 30 s each. Four independent replicates were measured. The data points were fitted to the model, assuming no photoinhibition (Webb et al., 1974). For further details, see Procházková et al. (2018b).

### Screening of UV Radiation and Blue-Green Light

Ultraviolet and blue light screening were assessed by chlorophyll fluorescence measurements (Bilger et al., 1997). Frozen and subsequently thawed cells of *Chlorella vulgaris* (SAG 211-11b) were used as a reference green alga from a non-exposed habitat. Algae (LP05) were sent frozen to University of Kiel, Germany, where they were stored at −20°C until measurement. The sample was thawed and applied onto a glass fibre pre-filter (Sartorius Stedim biotech, Göttingen, Germany). Chlorophyll fluorescence was excited from the sample using a Xe-PAM fluorometer (Walz, Effeltrich, Germany) with blue-green (half-bandwidth (HBW), 420–560 nm), UV-A (λ_max_, 366 nm; HBW, 32 nm), UV-B (λ_max_, 314; HBW, 18 nm) and red (λ_max_, 650 nm; HBW, 9 nm) measuring beams according to Pescheck and Bilger (2018). Ratios of fluorescence excited with blue-green light [F(BG)], UV-A [F(UV-A)] or UV-B [F(UV-B)] to that excited with red light [F(red)] were calculated. The measurements were conducted in three replicates. For comparison, a dense suspension of *Chlorella vulgaris* cells was measured the same time. In order to provide the same pre-treatment for these cells as for *C. krienitzii*, *Chlorella* cells were frozen for 2 h at −20°C and afterward rapidly thawed. *Chlorella* was cultivated in liquid culture medium in 400 mL culture vessels placed in a modified Kniese apparatus (Senger et al., 1972) under continuous aeration with filtered air. 3N BBM + V medium (modified BBM) (Starr and Zeikus, 1993) was used for the cultivation. The culture was grown on a 14:10 h light/dark cycle at 135 μmol photons m^–2^ s^–1^ of white fluorescent light at room temperature. Values of fluorescence excitation ratios of *C. krienitzii* were compared with those of *Chlorella vulgaris* cells using Student’s *t*-test with SigmaPlot (Systat Software, Erkrath, Germany).

### Pigment Analysis

For characterisation of carotenoids and chlorophylls (sample LP05), lyophilised cells were broken with liquid nitrogen in a mortar with a pestle, extracted with organic solvents and analysed by HPLC (Agilent 1200 ChemStation) equipped with a YMC C30 column and a diode array detector set at 450 nm in the same manner as described in Procházková et al. (2019b). Pigment ratios (w/w) were used because dry mass of cells as a reference could not be obtained due to the presence of particles.

### Lipid Extraction and Fatty Acid Methyl Esters Analysis (FAMEs)

The extraction procedure was based on the method of Bligh and Dyer (1959), and elution was done from a Sep-Pak Vac Silica cartridge 35cc (Waters; 10 g normal-phase silica) by chloroform (neutral lipids), acetone (glycolipids), and methanol (phospholipids) according to Saunders and Horrocks (1984). All classes of lipids were saponified overnight in 10% KOH in methanol at room temperature. The structures of fatty acid methyl esters analysis (FAMEs) were confirmed by comparison with Gas Chromatography/Mass Spectrometry retention times and fragmentation patterns with those of standard FAMEs (Supelco, Prague) (Dembitsky et al., 1993; Řezanka and Dembitsky, 1999). Procedures were described in more detail in Procházková et al. (2018b).

## Results

### Habitat Conditions

**FIGURE 1:**
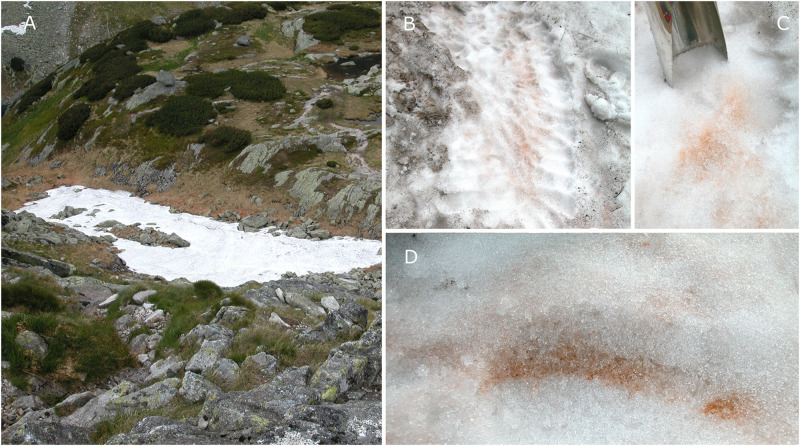
Overview of a sampling site in the valley Za Mnichem, the High Tatras, Poland, with a bloom of *Chloromonas krienitzii*. The snow field was located on a modest slope in a field depression **(A)** and contained superficial “black snow” (likely airborne mineral dust) **(B)**. Before cell harvest, to avoid a high load of mineral particles in a sample, the surface snow was removed **(C)**. Orange spots were visible at the snow surface **(B)**, but the highest algal cell density was about 3–5 cm below **(D)**.

In the High Tatras, the Sarntal Alps and the Northern Pindus mountain range, spots of orange snow were found in June and July at elevation from 1862 to 2240 m a.s.l., all of them situated above timberline (Table 1). In the Rocky Mts., the orange spots appeared as insertions in a large red snowfield dominated by *S. nivaloides* at 3540 m a.s.l. In the Polish High Tatras, the virtually monospecific orange snow was present at the surface, but the most intense bloom was about 3 to 5 cm below (LP05 sample). At this site, the surface was dominated by “black snow” (caused by inorganic particles) typical for this region (Kol, 1966). Although this blackish snow was removed before harvest, impurities were still present in the sample collected in subsurface layers (Figure 1). In the moment of harvest of the field cysts, a slightly acidic pH and low electrical conductivities were typical for the meltwater at all localities (Table 2).

**TABLE 2:** Abiotic habitat parameters and cyst sizes of *Chloromonas krienitzii* field samples.

**Sample**	**Species**	**EC**	**pH**	**SWE**	**Cells per mL meltwater**	**Cell size (μm)**
Saddle2	*S. nivaloides*, *C. krienitzii*	3	5.5	–	–	25.6−34.2 × 22.9−26.6 (2)
1SAR	*C. krienitzii*	–	–	–	–	22 ± 3; 15−27 (40)
LP05	*C. krienitzii*	6.8	5.2	53.8 ± 1.9	45 900 ± 4600	26 ± 1.8 × 21 ± 1.6; 22.9−29.4 × 18−24.3 (31)
WP189	*C.* cf. *nivalis*, *C. krienitzii*	8.1	6.2	–	(35 500); 850	21.6−25.7 × 22.3−24.4 (4)
WP190	*C. nivalis* subsp. *tatrae*, *C. krienitzii*	5.8	6.8	–	(15 250); 2050	23.9−30.4 × 23.3−28.1 (7)
WP191	*C. krienitzii*	–	–	–	–	24.9 ± 2.1 × 22 ± 1.7; 20.9−29.6 × 18.3−25.1 (33)
Pind19	*C. krienitzii*	–	–	–	–	33 ± 1.3 × 29.1 ± 1.5; 32.1−36.5 × 27.1−31.6 (10)

*Electrical conductivities (EC) (μS cm^–1^), pH of meltwater, the snow water equivalent (SWE) (%), maximum population density ± standard deviation (SD), average cell sizes [μm] and cell size ranges (with *n* = number of cells measured) are shown. In case *C. krienitzii* was a minor contribution in a bloom of other snow alga (e.g., *Chloromonas* cf. *nivalis*, *Chloromonas nivalis* subsp. *tatrae*), cell counts of the latter are shown in brackets.*

### Cell Morphology and Autofluorescence

**FIGURE 2:**
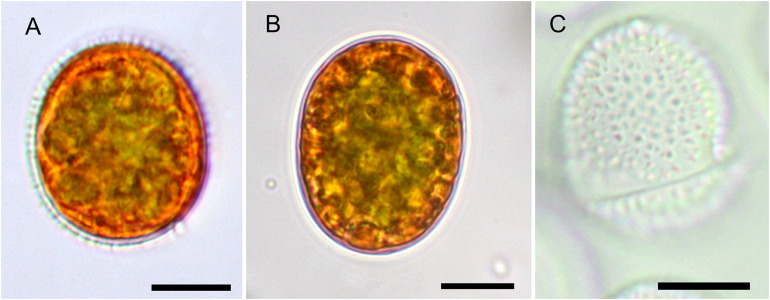
The Light microscopy of *Chloromonas krienitzii* field cysts. **(A,B)** Median view, showing cytoplasm with peripheral, orange astaxanthin deposits and an axial, greenish chloroplast. **(A)** Prominent secondary cell wall spikes still present. **(B)** Smooth cell that had shed the outer envelope **(C)**, the latter possessed characteristic, short spikes (<1 μm). Scale bar 10 μm.

**FIGURE 3:**
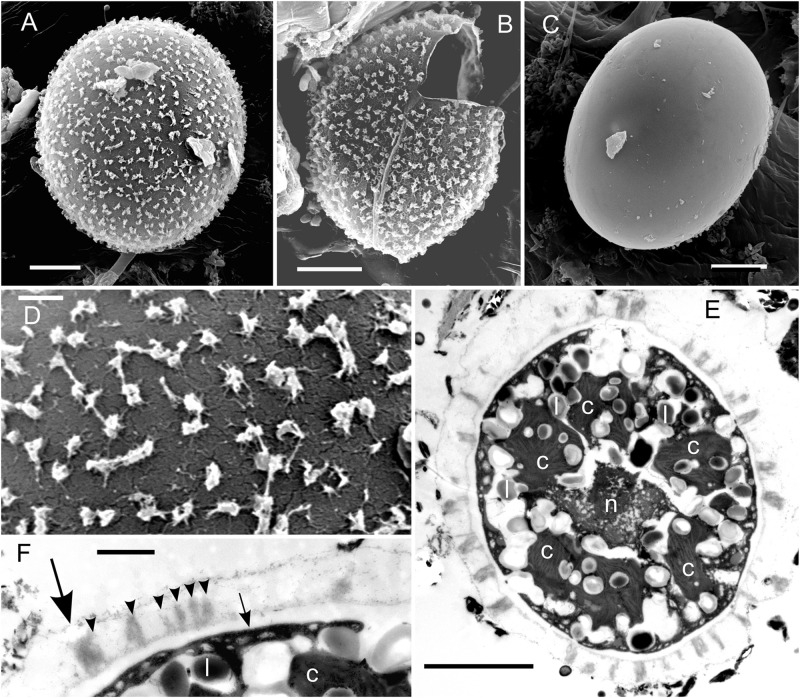
EM of *Chloromonas krienitzii* field cysts from the High Tatras (LP05). **(A–D)** SEM. **(A)** A cyst with spiky surface structures. **(B)** Outer (putative secondary) cell wall with spines after shedding. **(C)** Remaining (putative tertiary) smooth cell wall. **(D)** Detail view of the spiky surface of the secondary cells wall. **(E,F)** TEM of cells fixed by acidic lugol solution. **(E)** The chloroplast, sectioned into several discs (c), surrounding the central nucleus (n) and abundant lipids bodies (l) **(F)** Detail view of cell wall layers, transient primary cell wall (large arrow), spikes as a part of the secondary cell wall (arrowheads), smooth tertiary cell wall (small arrow). Scale bars 5 μm for panels **(A–C,E)**; scale bars 1 mm for panels **(D,F)**.

**FIGURE 4:**
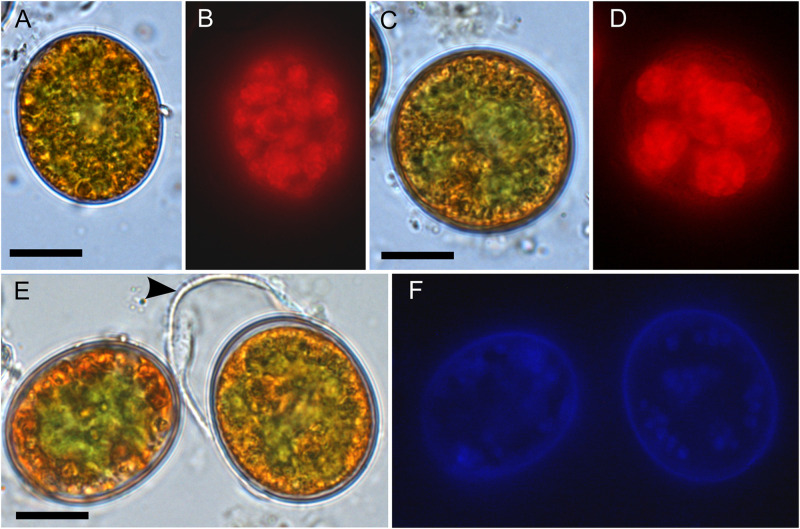
Bright field vs. fluorescence LM comparison of *Chloromonas krienitzii* field cysts. Chlorophyll autofluorescence in red **(B,D)** showing the chloroplast sectioned into several discoid parts, with are otherwise partly obscured **(A,C)**. The tertiary cell wall exhibited blue, UV A-induced autofluorescence **(F)**, indicating the presence of putative UV-absorbing compounds. In contrast, the outer, secondary spiky wall (arrowhead) exhibited no autofluorescence (compare **E,F**). Scale: 10 μm.

In the High Tatras, the maximal population density was 45,900 ± 4,600 orange cysts mL^–1^ meltwater was recorded (LP05). In the light microscope, blooms consisted of orange, egg-shaped cells with mean sizes of 25.1 ± 3.9 μm × 22.6 ± 3.1 μm (Figures 2–4). Cells still had their outer envelope (Figures 2A, 3A), were in the process of shedding their outer envelope (a cell on the right in Figure 4E) or this envelope was just shed (Figures 2C, 3B). This outermost ephemeral cell wall possessed many characteristic short protrusions (Figures 3D,F), in contrast to the persisting smooth cell wall surface (Figures 2B, 3C). The cytoplasm contained large numbers of peripheral, orange lipid bodies (Figures 1A, 3E, 4A,C), presumably deposits of the carotenoid astaxanthin which obscured the plastid morphology. Using chlorophyll autofluorescence, the chloroplast shape was revealed as axial and being sectioned into several discoid parts (Figures 4B,D). Remarkably, the remaining secondary cell wall exhibited a blue autofluorescence under UV-A exposure (Figure 4F). In contrast, vegetative flagellates of the strain (see below) had no wall UV-autofluorescence (data not shown).

### Comparative Analysis of ITS2 rDNA

**FIGURE 5:**
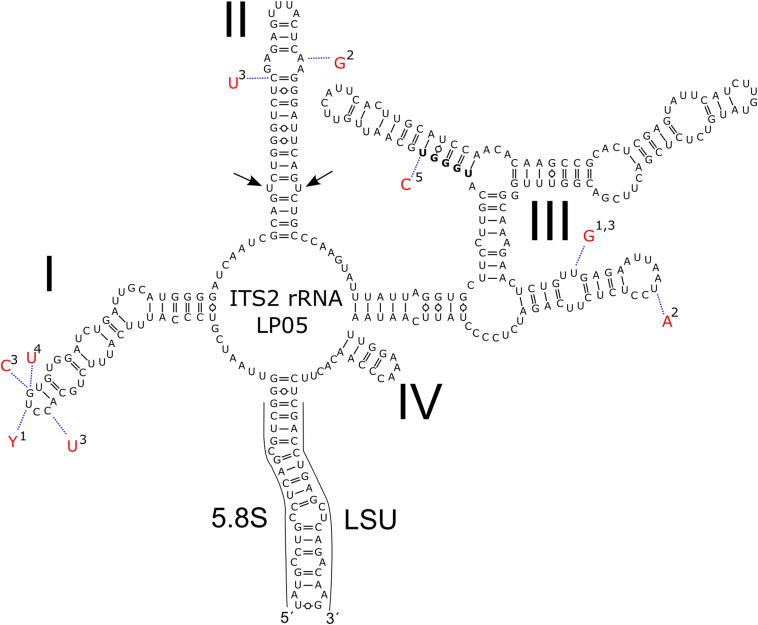
Intraspecific variability in the secondary of ITS2 rDNA transcripts of *Chloromonas krienitzii*–comparison between field-cyst collected from different localities (LP05 [MW136658], 1-1SAR [MW139362], 2-WP191 [MW139361]; this study), the type strain (3-GsCl-54 [LC012756]; Matsuzaki et al., 2015) and published sequences of field cysts (4-Gassan-A [LC012753], 5-Hakkoda-2 [LC012755]; Matsuzaki et al., 2015). The marker sequence differed by 1–4 bp amongst the samples. Helices are labelled with Latin numbers: I–IV. Nucleotide differences of the field samples are described in red outside the structure and linked by dotted lines. No compensatory base change was found. Note the U–U mismatch in helix II (arrow).

Regarding the variable marker ITS2 rDNA, cells of all blooms (LP05, 1SAR, WP191) were genetically identical with the type strain of *C. krienitzii* (GsCl-54) from Japan, except for one to four nucleotide positions (out of 288 bp). The different nucleotides were on a single strand only, thus no compensatory base change was found. The cysts in other samples collected in this study (Table 2) were used for the light microscopy observation only. Taking into an account these molecular findings together with the consistent morphology of the field sampled cysts and their identical type of habitat, all investigated samples were regarded to belong to this species (Figure 5).

### Life Cycle Studies

**FIGURE 6:**
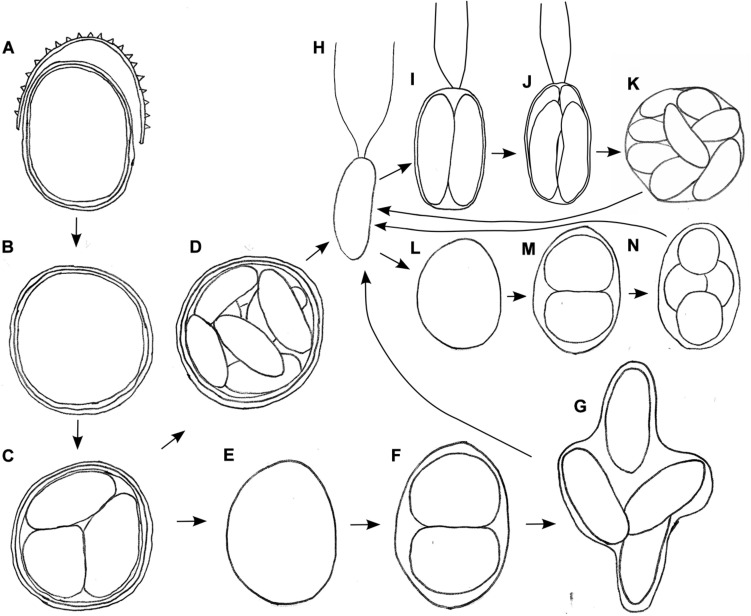
Schematic, putative parts of the life cycle of *Chloromonas krienitzii*, based on combined observation of two field samples (LP05, WP191) kept in original meltwater **(A–H)** and vegetative flagellates **(H)** transferred into 0.6 N BBM medium **(H–L)** at laboratorial conditions. **(A)** Cyst from the field shedding outer, spiky wall. **(B)** Remaining cysts with smooth wall (resembling *Cryodactylon glaciale*). **(C)** Meiosis and cyst germination resulting in four daughter cells (three shown). Subsequently, two independent ways of development were observed: **(D)** Mitotic division (up to 16 cells per cyst). **(E)** Growth of released daughter cell and **(F,G)** mitotic divisions. **(H)** Vegetative flagellates are elongate- kidney or elongate-bean shaped. Then, two ways of asexual reproduction via zoospore formation were found, either via elongated **(I–K)** or spherical stages **(L–N)**, in both cases two to sixteen daughter cells were produced within the parental cell wall. Not shown: gamete fusion to form a new zygote, respectively a cysts.

The aim of life cycle observation was to reconstruct the fate of the bloom cysts. The shedding of the spiky, outermost cell wall was observed for cysts in the field (WP191, LP05) and later in the lab as well (Figure 6A). In course of this life cycle development, the remaining cells with smooth wall and orange pigmentation resembled a different snow alga, *Cryodactylon glaciale* (Figure 6B), instead of initial spiky cells traditionally identified as *Chloromonas* cf. *brevispina*. After 5 months of reproductive inactivity in original meltwater in the lab, these smooth cysts had cleaved into 4 to 16 daughter cells. Concurrently, the intracellular pigmentation of sporangia turned from orange to green (Supplementary Figure 2A and Figures 6C,D). Approximately 3 weeks later, the cysts germinated, i.e., vegetative green thickened cells (Figures 6E–G) or elongate bean to kidney shaped flagellates were released (Figure 6H). Two morphologic versions developed, either elongate (Figures 6I–K) or spherical stages (Figures 6L–N). No sexual reproduction was observed for this strain. Still, the formation of a new generation of cyst stages was occasionally observed at lab conditions (Supplementary Figure 2H).

### UV and Blue Light Protection of the Cysts

**FIGURE 7:**
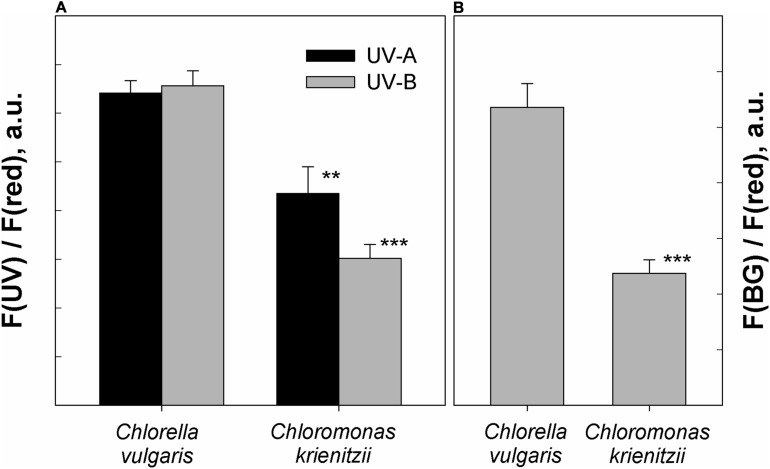
Protection against UV and blue light as determined by chlorophyll fluorescence measurements. **(A)** UV-A and UV-B fluorescence excitation ratios (arbitrary units) of frozen and subsequently thawed *Chlorella vulgaris* cells and *Chloromonas krienitzii* cysts (LP05). **(B)** Blue-green to red fluorescence excitation ratios (arbitrary units), indicating competition for excitation light between chlorophyll and carotenoids. Error bars show standard deviation, *n* = 3. Significant differences were calculated using Student’s *t*-test. **p < 0.01; ***p < 0.001.

In the fresh state, *Chlorella vulgaris* cells showed the same fluorescence excitation ratios as isolated chloroplasts from *Arabidopsis thaliana* (100% transmittance standard, data not shown). Since *C. krienitzii* cells were also frozen and thawed, its data may compare better to those of the frozen *Chlorella vulgaris* cells. Compared to *Chlorella vulgaris*, *C. krienitzii* showed a 25 to 35% higher UV-A screening (*p* < 0.01) while the screening of UV-B radiation was 40–50% higher (*p* < 0.001) (Figure 7A).

The astaxanthin of the snow alga shows strong absorption in the blue-green spectral region and should accordingly compete for light absorption with chlorophyll. Indeed, fluorescence excitation in the blue-green wavelength region (HBW 420–560 nm; Bilger et al., 1997) was reduced by about 50% as compared to astaxanthin-free *Chlorella* (*p* < 0.001) (Figure 7B).

### Photosynthesis

**FIGURE 8:**
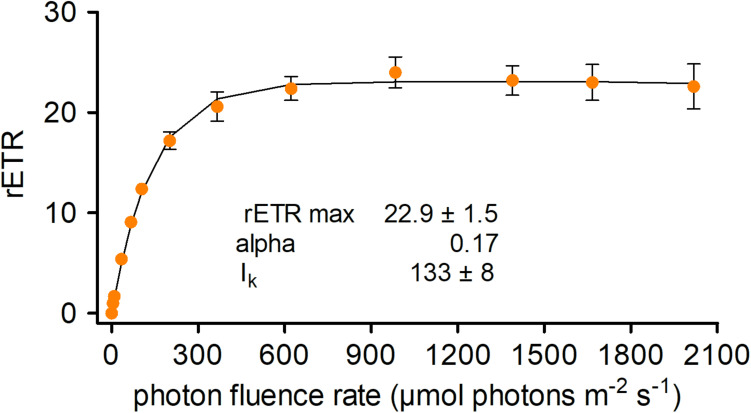
Photosynthetic rapid light curves of *Chloromonas krienitzii*. The effect of increasing photon fluence rates (*x*-axis) on the relative electron transport rate (rETR) (*y*-axis) of chloroplasts was measured for field-collected cysts (sample LP05) (*n* = 4, ±SD). The data points were fitted to the model, assuming no photoinhibition (Webb et al., 1974).

The photosynthetic performance of *C. krienitzii* field samples was tested at different irradiance levels and rapid light curves were generated. The cysts were not dormant in terms of photosynthesis, as indicated by ETR (Figure 8). No decline of activity was noticed up to 2100 μmol PAR m^–2^ s^–1^. Cells showed an alpha value of 0.17, a relative ETR_*max*_ of 22.9 ± 1.5, and an I_*k*_ value of 133 ± 8 μmol PAR m^–2^ s^–1^.

### Pigment and Fatty Acid Composition

**FIGURE 9:**
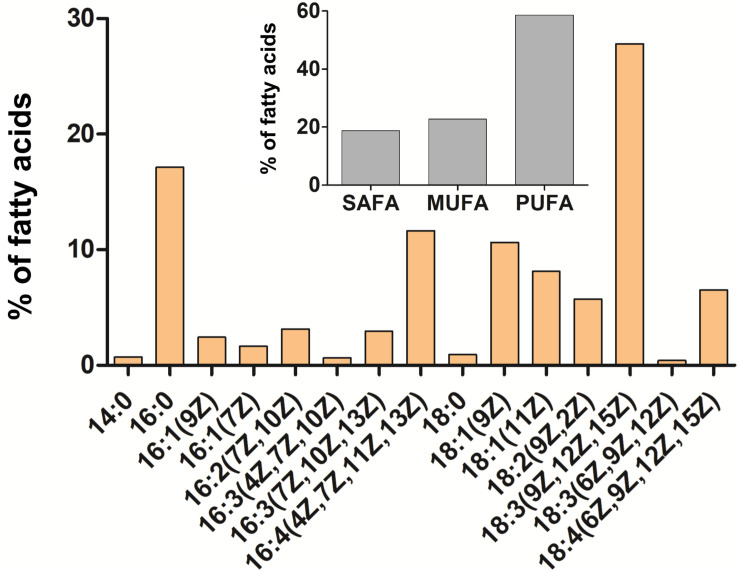
Cellular fatty acid composition of *Chloromonas krienitzii* cysts (LP05) in [%] of total fatty acids. The relative proportion of saturated (SAFA), monounsaturated (MUFA), and polyunsaturated (PUFA) fatty acids is given in the inset. The figure shows only fatty acids that had abundances greater than 0.1% of total fatty acids.

The orange pigmentation of cysts of *C. krienitzii* was caused by astaxanthin. Its abundance and those of other pigments were calculated in reference to chlorophyll *a* (Supplementary Table 1). Astaxanthin comprised 28% of all pigments (LP05). In the chromatogram, it occurred as several peaks with identical absorption spectra, all of them likely ester derivatives (data not shown). Chlorophylls (a and b) comprised 61% of all pigments, primary (plastid) carotenoids represented 11%. The overall ratio of astaxanthin to chl-*a* was 0.4. In contrast, the laboratory strain stayed green (data not shown). The relative content of FAs (in percentage of total fatty acids) of *C. krienitzii* field cysts (LP05) is shown in Figure 9. FAs with C14 to C18 were found. Cells showed high levels of PUFAs (58.6% of total fatty acids), whereas the content of saturated acids (SAFAs) did not exceed 18.7% (mainly palmitic acid, 16:0, 17.1%). The main monounsaturated fatty acid (MUFA) was oleic acid (18:1 (9Z), 10.6%). The dominant PUFA was linolenic acid [18:3 (9Z, 12Z, 15Z), 27.8%], followed by hexadecatetraenoic acid [16:4 (4Z, 7Z, 11Z, 13Z), 11.6%].

## Discussion

### Geographical Distribution of Snow Algae

Direct sequencing of monospecific algal blooms facilitates species-specific biogeographic conclusions (Procházková et al., 2019a). Individual species of snow algae can either show a cosmopolite distribution (e.g., *S. nivaloides*, Segawa et al., 2018; Procházková et al., 2019a) or their known occurrence is rather geographically limited (e.g., *Chloromonas polyptera*, Remias et al., 2013; *Sanguina aurantia*, Brown and Tucker, 2020). *C. krienitzii* seems to be in-between: It was initially described only from Japan (Matsuzaki et al., 2015) and subsequently found in Europe and North America as well (this study, Engstrom et al., 2020). Likely, the morphologically striking cysts have been observed many times elsewhere, but were determined as *Chloromonas* cf. *brevispina* due to the spiky wall surface, and molecular protocols were not available during older studies. For example, Hoham et al. (1979) described snow algal cysts similar to *C. krienitzii* in the United States. This work gives the first records of *C. krienitzii* for Europe, namely in the High Tatra Mts (Poland and Slovakia) and the Alps (Italy), which is based both on molecular and morphological data. Furthermore, cysts with the same morphology were found in Greece (this study) and previously reported from Sierra Nevada (Spain) (see Figures 23–25 in Cepák and Lukavský, 2012) and Stara Planina Mts. (Bulgaria) (see Figures 50–55 in Lukavský and Cepák, 2010). *C. krienitzii* has not been found in polar regions or the Southern hemisphere yet.

### Species Distribution Along the Gradient of Elevations

The blooms occurred at open sites with elevation ranging from 1862 to 3500 m (this study), but earlier studies reported *C. krienitzii* to be predominant in clearings or sites with sparse trees around 1200 m (Engstrom et al., 2020) or at open sites in broad-leaf forest around 880 m elevation (R. Matsuzaki–pers. comm., Matsuzaki et al., 2015). Similarly, such a wide gradient of elevation was described for the occurrence of orange to pinkish snow caused by *Chloromonas hindakii* (Procházková et al., 2019b). Apparently, snow algae of the genus *Chloromonas* can thrive in a large range of irradiation levels (exposed to semi-shaded sites). In contrast, *S. nivaloides*, the probably most widespread cryoflora species (Procházková et al., 2019a), is restricted to exposed habitats (Remias et al., 2005). The slightly acidic pH and low meltwater conductivities where *C. krienitzii* was found correspond to similar values reported for many other chlorophycean snow algae (Hoham and Remias, 2020).

### Colours of Snow Bloom in the Context of *Chloromonas brevispina*-Like Species

Using molecular data, Matsuzaki et al. (2015) proved that cysts which were morphologically determined as *C. brevispina* are caused by several independent lineages, which are in several cases not taxonomically described yet. In Washington State (United States), mature zygotes *C. brevispina* were reported to develop yellow, orange or pink snow discolouration, or to remain green if found several centimetres below the snow surface (Hoham et al., 1979). In this study, solely orange cells were observed for *C. krienitzii* in the field samples. Different stages of pigmentation (reflecting the changing ratio astaxanthin to chlorophyll) were noticed for other representants of *Chloromonas* cf. *brevispina* (*C. brevispina* DL09 in the Austrian Alps, Lutz et al., 2019, usually causing green snow, while the pink version was observed in late summer, Remias–pers. obs.).

### Different Morphological Stages in Course of Life Cycle

Combined field and lab observations have provided insights to the life cycle of several snow dwelling *Chloromonas* (e.g., Hoham, 1975; Hoham and Mullet, 1977; Hoham et al., 1983). For *C. krienitzii*, the transition from a cyst to vegetative stages was presented here for the first time. As for other *Chloromonas* species, the “gametic membrane,” like in the case of *C. krienitzii*, may remain surrounding the developing zygote, later it is expanded, gelatinises and is eventually shed (Procházková et al., 2018a). Smooth-walled cyst stages with partially orange cell compartments are difficult to be determined to the species level by light microscopy. Formerly, these cells were regarded as the independent species *Cryodactylon glaciale* (Chodat, 1921; Kol, 1968). We never observed shedding of secondary cell walls for other *Chloromonas* cf. *brevispina* species; in fact, they keep their spiky cell walls throughout the season (data not shown). During cyst germination, the number of daughter cells in *C. krienitzii* was always four, indicating that this process is probably meiotic. Concurrently, intracellular astaxanthin redistribution between daughter cells was noticed. It was followed by astaxanthin decomposition in these daughter cells (Supplementary Figure 2A). This can be interpreted as an adaptation to low-light conditions deep in the snow after germination during springtime, where secondary carotenoids would impair the photosynthetic performance by shading the chloroplast.

In the culture of *C. krienitzii*, the dominant vegetative stages were bean-shaped flagellates, however, spherical cells were regularly observed as well. This roundish shape is regarded as not being very common for *Chloromonas*, still it has been observed in several strains, and occasionally in the field (Hoham et al., 1979; Procházková, Remias–pers. obs.). The asexual reproduction of vegetative strain formed by flagellates for *C. krienitzii* was described previously (Matsuzaki et al., 2015). This corresponded with our finding that just before the first cell division, the protoplast rotated, and the parental contractile vacuoles moved to the equator of the parent cells (Supplementary Figure 2C, compare with Figure 11 in Matsuzaki et al., 2015). Without such observation, one may have a misleading impression that the strain contains two unrelated flagellates–thin elongate ones (i.e., not dividing at the given moment) and thickened ones (i.e., prepared for cell division). During this study, packages of two to four flagellates still inside the parental cells were occasionally observed (analogy can be seen in Figures 9, 10 in Hoham et al., 1979). The flagellates of *C. krienitzii* were “backward”-swimmers, meaning that during the cell movement the flagella are in the rear (Supplementary Video 1). In this study, sexual reproduction (e.g., Matsuzaki et al., 2020) was not observed.

### Photosynthesis at Changing Light Conditions

Life at high alpine sites requires adaptation to extreme abiotic factors like temperature, visible and UV radiation and to their significant diurnal variations. Fluorometric measurements showed that the *C. krienitzii* cysts were not dormant in terms of photobiology similarly to other snow algal species (Remias et al., 2013; Procházková et al., 2018b). The photosystem II was well adapted to high levels of irradiation and showed no sign of photoinhibition up to 2100 μmol PAR m^–2^ s^–1^. In mid-latitude regions, cells are subject to such irradiances at the snow surface on sunny days (Gorton and Vogelmann, 2003). Nonetheless, relatively high alpha value indicated that this species was shown to perform well also under low light conditions. This corresponds to the localisation of most cells 3 to 5 cm below snow surface.

### Cellular Adaptation to Elevated Light Levels at Alpine Conditions

The apparent ability of the cysts to cope with high light conditions is a result of an interplay of several cellular adaptations. The fluorescence excitation ratio of blue-green light (BG, 420 to 560 nm; Bilger et al., 1997) to red light (R, 650 nm) was explored, which gives an indication on the competition between carotenoids and chlorophyll for excitation light (Nichelmann et al., 2016). In *C. krienitzii*, this ratio was significantly lower when compared with a non-cryoflora *Chlorella* reference. Spectral data provided evidence that carotenoids are the compounds which were responsible in *A. thaliana* for the observed changes in the fluorescence excitation efficiency between chloroplasts adapted to different light levels (Nichelmann et al., 2016). In *C. krienitzii* cysts, the significant reduction of the F(BG) to F(R) fluorescence excitation ratio may be attributed to intracellular deposits of carotenoids since the spectrum of the BG excitation beam covers roughly the visible absorption of astaxanthin. Light must pass through a layer of this pigment at the cell periphery before it reaches the centrally located chloroplast (Figure 2B). In addition, a high pool size of violaxanthin (V), antheraxanthin (A), and zeaxanthin (Z) (V-cycle) could have contributed to the reduction of this ratio. However, the carotenoids of the VAZ cycle were present only in a comparatively low concentration (Supplementary Table 1). Thus, a screening function of astaxanthin was much more probable than one of the V-cycle xanthophylls. If algae display a similar adjustment of the VAZ pool size to irradiance as higher plants (see e.g., Nichelmann et al. (2016) and citations therein), the comparatively low pool size observed here indicates that the photoprotective nature of the outer carotenoid layers was quite effective. The cellular concentration of astaxanthin varies depending on the individual species and stage within the life-cycle of chlamydomonadacean snow algae (Hoham and Remias, 2020). While vegetative cells of *C. krienitzii* produced no astaxanthin, cysts had an astaxanthin to chl-a ratio of 0.4. Similar levels were found for *Chloromonas nivalis* cysts from the Austrian Alps (Remias et al., 2010). In contrast, species causing red snow at exposed sites (*Chlainomonas* sp. and *S. nivaloides*) have significantly higher ratios (Remias et al., 2005; Procházková et al., 2018a).

### Ultraviolet Radiation

High levels of short wavelength irradiation are typical for alpine environments. Harmful effects of UV on ultrastructure and metabolism in algae were reviewed by Holzinger and Lütz (2006). They include destruction of chloroplasts and mitochondria, and are probably mitigated by adaptive structures likely related to the UV stress (in the case of cryoflora: intracellular lipid bodies and vacuoles containing secondary pigments or partially crystallised content, respectively) (Holzinger and Lütz, 2006). Cells growing deeper in snow are less exposed to UV stress, since transmittance drops more rapidly for UV than for VIS with increasing snow depth (Gorton and Vogelmann, 2003). On the other hand, the localisation of a cell within the vertical snow profile may change throughout the day due to snow melt processes. The smooth tertiary cell wall of *C. krienitzii* exhibited a blue autofluorescence, which indicates the presence of UV-absorbing protective polyphenolic compounds. In contrast, the glacial algae *Mesotaenium berggrenii* and *Ancylonema nordenskioldii* do not exhibit such UV induced cell wall autofluorescence (Remias-own observation). On the other hand, this cell wall signal was observed for other close relatives of *C. krienitzii*, e.g., *C. hindakii* and “*Scotiella cryophila*-K1” cysts (Procházková-own observation) which indicates that this strategy may be common for cyst stages for *Chloromonas*. Furthermore, the measurements of fluorescence excitation ratios confirmed a significant UV-B and UV-A screening capability of *C. krienitzii* (Figure 7). Interestingly, the UV absorbance spectrum of isolated cell walls from snow alga *Chlamydomonas nivalis* cells (Gorton and Vogelmann, 2003) resembled that of sporopollenin (Xiong et al., 1997). In the latter study, the UV-B tolerant algal species contained substantial amounts of acetolysis-resistant residues of sporopollenin, while the sensitive species contained little or no sporopollenin. Still, for methodological reasons, the nature of the UV screening substances in the cell wall of *C. krienitzii* cysts has not been determined yet. Although only a very limited portion of the living and fossil algae have been studied for the presence and composition of resistant cell walls (Versteegh and Blokker, 2004), the existence of sporopollenin in *C. krienitzii* walls would not be surprising since zygospores of a related green alga is known to contain such resistant macromolecules (*Chlamydomonas monoica*; VanWinkle-Swift and Rickoll, 1997). An intracellular UV protectant may be represented by astaxanthin. Interestingly, in red pigmented cell of *Chlamydomonas nivalis*, cytoplasmatic compounds absorbed UV more strongly than the cell walls (Gorton and Vogelmann, 2003). Indeed, astaxanthin has maximum absorbance in the visible region but still a significant capability in the UV-A region (Remias, 2012), which may become important at the high astaxanthin concentrations observed here.

### Low Temperatures

A further challenge, for living in melting snow, represent low temperatures around the freezing point. At such conditions, a major role in avoiding membrane rigidity is played by unsaturation of the fatty acids in membrane lipids (Morgan-Kiss et al., 2006). This may correspond with the detected high level of PUFAs in *C. krienitzii* cysts. The level of PUFA likely relates to the presence of well-developed chloroplasts. In addition to galactolipids in chloroplasts, highly unsaturated phosphatidylcholine was identified in *Chlamydomonas reinhardtii* (Vieler et al., 2007), and phosphatidylglycerol in *C. reticulata* (Lukeš et al., 2014). In this study, α–linolenic acid as the dominant unsaturated FA in cells of *C. krienitzii* was consistent with profiles of other *Chloromonas* species harvested from snow (e.g., Řezanka et al., 2014) or from those kept in nitrogen deficient medium (Spijkerman et al., 2012). The previous analysis of three major lipid groups in related *Chloromonas* species dwelling in snow showed that PUFAs are present in phospholipids, glycolipids as well in neutral lipids (Procházková et al., 2018b, 2019b). In this study, a total fatty acid profile was performed for field-collected cysts of *C. krienitzii*, so the specific allocation of these compounds remains open. At the cyst stage, the proportion of PUFAs in *C. nivalis* subsp. *tatrae* was nearly 70% in lipid groups associated with membranes (glycolipids, phospholipids, etc.), and it was twice lower in neutral lipids (Procházková et al., 2018b) which are likely deposited in cytosolic lipid bodies as storage products (Thompson, 1996). When cultivated at 1°C, vegetative flagellates of snow alga *C. hindakii* had almost as high relative PUFAs contribution in all the three lipids classes; PUFAs accounted for ∼64% in neutral lipids, ∼65% in phospholipids and ∼75% in glycolipids (Procházková et al., 2019b).

## Conclusion

To conclude, cyst stages of the snow alga *C. krienitzii* possess effective mechanisms for protection against harmful UV and excessive VIS radiation, because screening compounds are localised in both the remaining smooth cyst wall and in peripheral cytoplasmic compartments. The nature of the former compound is not known (sporopollenin?), the latter was shown to be astaxanthin. Absorbance spectra of cell wall extracts remain to be determined. Next, cells were photosynthetically active under ambient conditions and were not impaired at low or high irradiation levels. Furthermore, the results showed that the adaptation of this snow alga to low temperatures possibly included high levels of PUFAs, although future work should evaluate this hypothesis with more comprehensive analyses of distinguishing between membrane and storage lipids. The findings showed that *C. krienitzii* is more widespread than previously known, it occurs in the northern hemisphere without the Arctic region. Further cryoflora relatives in the *C. brevispina*-like complex are awaiting their characterisation once flagellate stages will be available (Nedbalová et al., 2008; Matsuzaki et al., 2015).

## Data Availability Statement

The datasets presented in this study can be found in online repositories. The names of the repository/repositories and accession number(s) can be found below: NCBI GenBank, accession nos: MW136658 (sample LP05), MW139361 (sample WP191), MW139362 (sample 1SAR).

## Author Contributions

LP and DR designed this study, conducted the independently light microscopy, prepared a draft of the manuscript, and collected the samples with independent assistance from HK. LP sequenced the field material with assistance of HK, wrote the manuscript with the edit and input of DR. LP was further responsible for PAM measurements and electron microscopy observations. DR performed the pigment analysis, fluorescence light microscopy, and cultivation assays. TŘ carried out fatty acid methyl esters analysis. WB carried out chlorophyll fluorescence assay and contributed with the relevant part. TŘ and LN edited the final manuscript. All authors discussed the results and contributed to the final manuscript.

## Conflict of Interest

The authors declare that the research was conducted in the absence of any commercial or financial relationships that could be construed as a potential conflict of interest. The reviewer EM is currently organising a Research Topic with one of the authors [LN]. The review process met the standards of a fair and objective review.
