# Supplementary material for: Cysts of the Snow Alga Chloromonas krienitzii (Chlorophyceae) Show Increased Tolerance to Ultraviolet Radiation and Elevated Visible Light
Source: Front Plant Sci. 2020 Dec 17;11:617250. doi: 10.3389/fpls.2020.617250 (PMC7773729; doi:10.3389/fpls.2020.617250)
Supplement: Supplementary file 1 [file Table_1.DOCX]

Supplementary Material

**
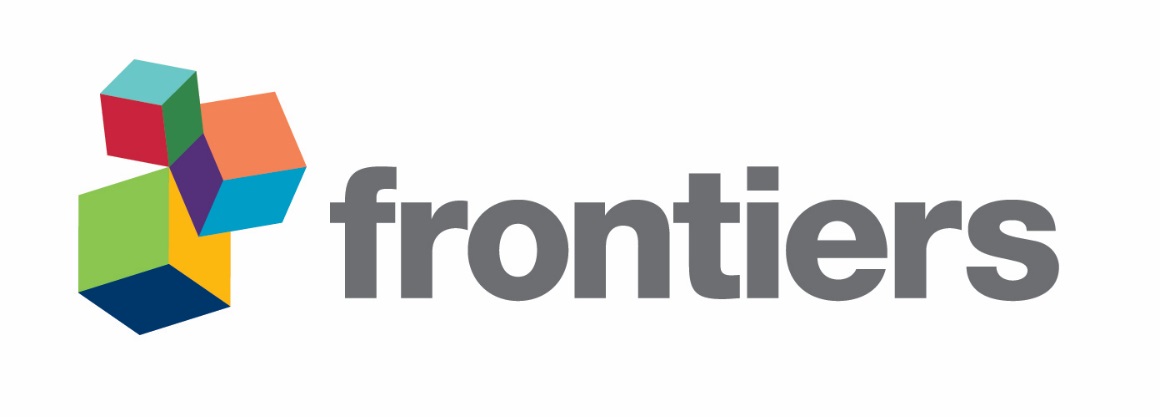
**

**
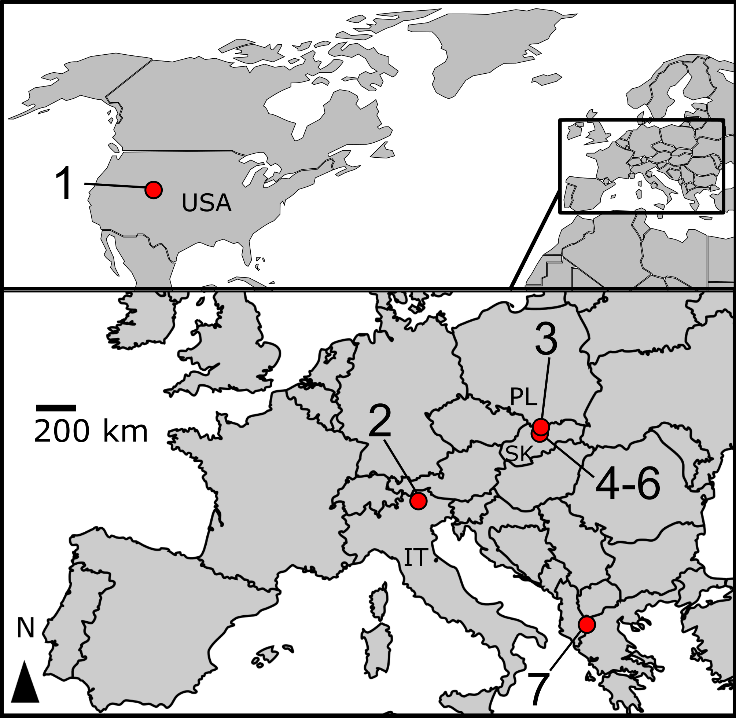
Supplementary Figure 1.** Snow sampling locations of *Chloromonas krienitzii* (red circles) in North America (above) and Europe (below), the numbers refer to those in the Table 1.

**
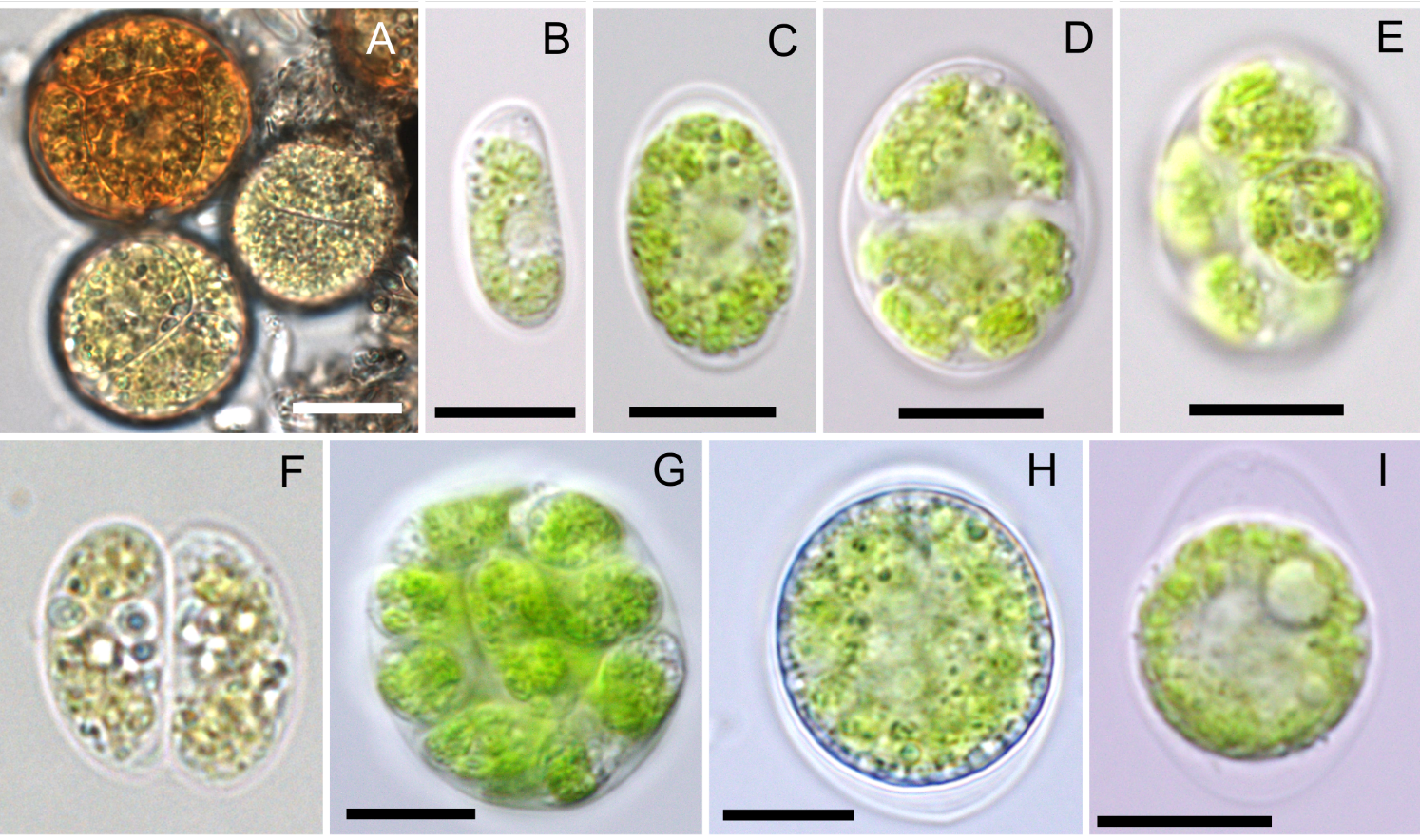
Supplementary Figure 2.** LM of selected *Chloromonas krienitzii* life cycle stages. (A) cyst germination and re-greening after prolonged exposure in original meltwater, (B) vegetative flagellates grown in (C-J) 0.6N BBM medium at lab conditions. (A) Putative meiosis resulting in 4 daughter cells. (B) Bean shaped vegetative flagellate. Then, there are two paths (I., II.) of development simultaneously: (I.) (C) grown vegetative cell and (D) mitotic division with (E) formation of spherical four zoospores within the parental cell wall. (II.) (F) asexual reproduction via formation of elongate zoospores within the parental cell wall, (G) up to 16 cells per autosporangium. (H) formation of a new cyst stage (rarely at lab conditions). (I) Uncommon cell stage with unknown position on the life cycle (planozygote?). Scale bar 10 µm.

**Supplementary Video V1.** (LP05_foursporangium flagellates.avi) showing the rotary movement of one quadrisporangium flagellates and motile backward swimmers of *Chloromonas krienitzii* LP05 strain. Duration 18 sec.

**Supplementary Table 1.** Relative content of pigments in ratios to chlorophyll a (=1; w/w) in a field sample of *Chloromonas krienitzii* from the High Tatras (LP05), determined by HPLC. Abbreviations: N&V, neoxanthin and violaxanthin; Ant, antheraxanthin; Lut, lutein; Zea, zeaxanthin; Chl *b*, chlorophyll *b*; Ast = astaxanthin plus astaxanthin derivatives (all-*trans*); asi, astaxanthin *cis*-isomers; n.d., not detected.

|  | N & V | Ant | Lut | Zea | β-car | Chl *b* | Ast | AsI |
| --- | --- | --- | --- | --- | --- | --- | --- | --- |
| LP05 | 0.062 | n.d. | 0.255 | 0.013 | 0.006 | 0.210 | 0.421 | n.d. |
